# Case Files from the University of California San Diego Medical Toxicology Fellowship: Neonatal Flecainide Toxicity from an Accidental Dosing Error

**DOI:** 10.1007/s13181-024-01018-9

**Published:** 2024-07-11

**Authors:** Justin Seltzer, Aaron Schneir

**Affiliations:** Division of Medical Toxicology, Department of Emergency Medicine, UC San Diego, 200 W. Arbor Dr #8676, 92103 San Diego, CA United States

**Keywords:** Flecainide, Pediatrics, Neonatology, Pediatric cardiology, Antidysrhythmic toxicity

## Case Presentation

*A twenty-two-day-old, 3.65 kg male, presented to the emergency department (ED) due to concern for potential accidental flecainide overdose. The patient had been delivered by caesarean section at 36 weeks’ gestation after recognition of in utero supraventricular tachycardia (SVT). The SVT resolved spontaneously after birth. Two weeks later, the infant presented to an outside ED for evaluation of emesis and rapid heart rate. At that time, he was found to have SVT that did not respond to three doses of adenosine, 0.1 mg/kg once followed by 0.2 mg/kg twice. He was transferred to the regional neonatal intensive care unit for higher level of care. On arrival, he was noted to have a heart rate of 300 beats per minute; pediatric cardiology felt this represented atrial flutter. Echocardiography at that time showed a diminished ejection fraction of 17%. Synchronized electrical cardioversion at 1 J/kg was then used to successfully treat the dysrhythmia, restoring normal sinus rhythm after a single shock.*

*During this hospitalization, amiodarone 9 mg/kg total and propranolol 2 mg/kg/day divided every 6 h were initially trialed at the recommendation of pediatric electrophysiology, along with a milrinone infusion due to the patient’s low ejection fraction. This regimen was ultimately unable to prevent recurrent episodes of SVT, which responded well to 0.2 mg/kg adenosine boluses. Consequently, oral flecainide 98 mg/m* ^*2*^ */day (10 mg) divided every 12 h and propranolol 2.4 mg/kg/day (2 mg) divided every 6 h were used and successfully maintained durable sinus rhythm.*

**Fig. 1:**
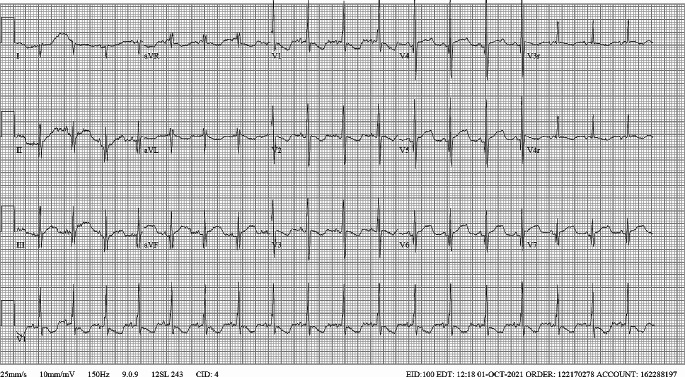
The patient’s most recent baseline ECG

*The patient was discharged on oral flecainide 10 mg twice per day, and oral propranolol 2 mg four times per day. An electrocardiogram (ECG) obtained prior to discharge (*Fig. 1*) on the appropriate doses of flecainide and propranolol, demonstrated a rate of 111 beats per minute, PR interval of 106 ms, QRS interval of 80 ms, and QTc interval of 454 ms. At that time, the serum flecainide concentration was 0.37 µg/mL (therapeutic range 0.20-1.00).*

### What is Flecainide and what is its Mechanism of Action?

Flecainide is classified as a Vaughan Williams class IC antiarrhythmic that is approved in the United States by the Food and Drug Administration for the prevention and treatment of supraventricular tachycardia, paroxysmal atrial fibrillation and atrial flutter, and ventricular tachycardia [1, 2]. Like all class I antiarrhythmics, flecainide exerts its therapeutic activity primarily through interactions with the cardiac sodium channel. Specifically, flecainide antagonizes fast inward voltage-gated sodium channels, slowing inward sodium movement during phase zero of cardiac depolarization. Flecainide has increased sodium channel binding at higher heart rates, a property known as “use-dependence.” Overall, this effect manifests as QRS prolongation on the ECG. Flecainide dissociates from the sodium channel slowly compared with many other antiarrhythmic sodium channel blockers. Flecainide also inhibits opening of the delayed rectifier potassium channels which manifests electrocardiographically as a prolonged QT interval [3].

## Case Continuation

*Four days following discharge, the patient’s mother recognized that the provided flecainide had a concentration (20 mg/mL) twice of that intended (10 mg/mL). The patient had received a total of eight doses when the parents contacted the patient’s cardiologist, who recommended ED presentation for evaluation despite being otherwise asymptomatic.*

*Two and a half hours prior to this presentation, his parents administered the correct total dose of 10 mg following recognition of the dosing error. The propranolol had been dosed and administered as intended.*

### Has Flecainide been Previously Implicated in Clinically Significant Pediatric Dosing Errors, and if so, why has this Occurred?

Flecainide dosing errors resulting in significant toxicity have been well described in the pediatric population. As flecainide has a narrow therapeutic index, toxicity may occur with modest dosing errors [4]. This is likely due to the lack of a commercially produced flecainide suspension, requiring compounding into a suspension from the adult tablet form [5]. As accurate dosing relies on correct preparation, labeling, storage, dosing instructions, and then ultimately dispensing, errors have occurred at every step in both inpatient and outpatient settings [5–10].

Several notable examples of flecainide’s narrow therapeutic index and potentially dangerous toxicity in very young pediatric patients have been reported in the literature. An 18-day-old received a two-fold dosing error (8 mg every eight hours instead of 4 mg every 8 h for four doses) due to a labeling error. Following the fourth dose, the neonate developed bradycardia without a palpable pulse. Following atropine administration, a wide complex tachycardia occurred that was successfully treated with sodium bicarbonate [2]. A 23-month-old was found to have a new bifascicular block following a single, doubled-dose [10]. A 48-day-old received nine doses of flecainide at 1.7 times the recommended upper pediatric dosing limit. Following the seventh dose, convulsions occurred, and following the ninth dose a wide complex tachycardia was recognized that resolved without intervention in three days [11]. A four-month-old received six times the intended dose as a result of a syringe measurement error for multiple days. A sustained wide complex tachycardia with hemodynamic instability developed and was successfully treated with venoarterial extracorporeal membrane oxygenation (VA-ECMO) [8]. A single, five-fold dosing error administered to a 7-month-old as a result of syringe measurement error caused pulseless ventricular tachycardia that was successfully treated with sodium bicarbonate boluses, lipid emulsion, and VA-ECMO [7].

In utero SVT can also be managed via transplacental delivery of maternally ingested flecainide. ECG findings consistent with flecainide toxicity have been noted at birth following maternal flecainide administration, even if the mother shows no electrocardiographic signs of flecainide toxicity and has a therapeutic serum flecainide concentration [12, 13].

### Even when the Correct Dose of Flecainide is Administered, Adverse Effects can Occur. What are Potential Reasons for this?

Ventricular dysrhythmias attributed to flecainide have been described with flecainide even when the correct therapeutic dosing is administered [14]. This is particularly true in patients with myocardial ischemia and ventricular dysfunction, two settings in which flecainide is not recommended [15].

In vivo, a majority (~ 70%) of flecainide is metabolized to the less active metabolite meta-O-dealkylated flecainide, primarily by cytochrome (CYP) 2D6. The active metabolite is subsequently metabolized to an inactive metabolite and renally cleared. The remaining flecainide is renally cleared and excreted unchanged in the urine [3].

CYP2D6 polymorphisms are found throughout the general population and can result in a wide range of metabolic profiles. Poor metabolizers have no CYP2D6 function and intermediate metabolizers have approximately 25 to 50% function. Ultrarapid metabolizers, who carry a CYP2D6 gene duplication, metabolize substrates faster than normal metabolizers [16, 17]. Substantial differences in flecainide metabolism have been established between those who are extensive metabolizers versus those who are poor metabolizers [18, 19]. For example, toxicity developed in a neonate who was on appropriately-dosed and -administered flecainide, which was later attributed to being a CYP2D6 intermediate metabolizer [20]. Salient to neonatal patients specifically, CYP2D6 becomes active within hours or days after birth, and its maturation is thought to be complete at about one year of life [21]. A genetic polymorphism should be considered following unexpected toxicity or high plasma concentrations from therapeutic dosing [22].

CYP2D6 inhibition, which can occur from multiple agents including amiodarone, paroxetine, and flecainide, should also be considered when unexpected toxicity occurs [23–27]. Our patient was only on flecainide and propranolol at the time of presentation. While propranolol is a substrate of CYP2D6, it is not a clinically significant CYP2D6 inhibitor [17, 28].

Beyond pharmacogenetics, general impairment of hepatic metabolism and/or renal clearance can lead to dangerous accumulation of even therapeutic doses, resulting in toxicity [3, 29–35]. Poor metabolizers with renal dysfunction are at significant risk [36].

## Case Continuation

**Fig. 2:**
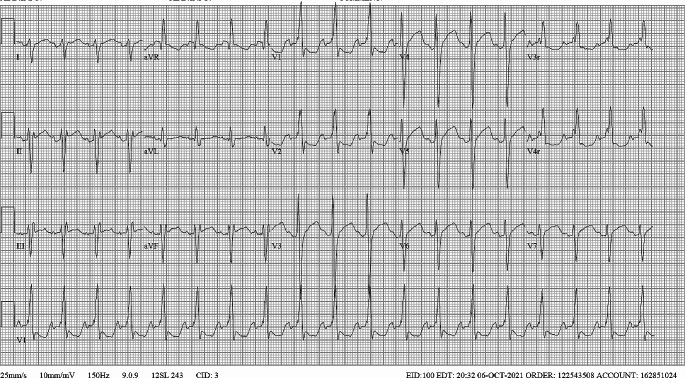
ECG obtained in the ED at the time of presentation

Upon hospital presentation after the dosing error was recognized, the patient appeared well. Vital signs and physical examination were normal. An ECG (Fig. 2) demonstrated a heart rate of 113, PR interval of 118 ms, QRS interval of 138 ms, and QTC interval of 513 ms. Laboratory testing was notable for a serum potassium of 5.7 mmol/L, magnesium 1.9 mg/dL, and calcium 11.3 mg/dL. Renal function and liver enzymes were within normal ranges. A flecainide concentration was drawn, but results were not rapidly available as it was a send-out test.

### Is the Patient’s Presentation Consistent with Flecainide Toxicity and with the Described Dosing Error?

The patient’s ECG is consistent with flecainide toxicity. Electrocardiography is the primary diagnostic tool for demonstrating the cardiotoxic effects of flecainide, regardless of clinical symptoms [3, 10, 37]. The ECG shown in Fig. 2 demonstrates interval prolongation of the PR, QRS, and QTc intervals from therapeutic baseline (Fig. 1). These are findings consistent with the known electrophysiologic effects of flecainide [3, 38, 39]. It is valuable to recognize that prolongation of the PR, QRS, and QT intervals also occur at therapeutic dosing and the degree of prolongation is expected to correlate with increasing concentrations of flecainide [38].

The clinical presentation associated with flecainide toxicity is nonspecific and largely attributed to its cardiotoxicity. Bradycardia with hypotension and clinical signs of shock, such as lethargy or obtundation, are well described with severe toxicity, as are seizures. No pediatric specific features have thus far been described [2, 7, 8, 10–12, 35, 37, 40–43]. Less severely poisoned patients may report nausea and vomiting, dizziness, headache, and visual disturbances [42]. In this case, no discernable clinical manifestations beyond ECG changes were noted.

On review of potential alternative explanations to the described dosing error for the observed electrocardiographic changes, none were likely. The patient had been taken off of amiodarone prior to flecainide initiation and showed no signs of a drug interaction prior to discharge. Metabolic impairment of CYP2D6 would be expected to manifest electrocardiographic abnormalities more rapidly after flecainide initiation. No clinically significant electrolyte abnormalities were noted at the time of presentation. And finally, the patient had no known exposure to CYP2D6 inhibitors. The patient was breastfeeding and, to our knowledge, his mother was not on medications that could have passed to the patient via breast milk. Consequently, the described dosing error is the most plausible explanation for the patient’s ECG abnormalities.

### Does Activated Charcoal Bind to Flecainide, and should it be Administered in this Case?

An adult volunteer study of therapeutic flecainide ingestions showed that activated charcoal administration decreased flecainide absorption and, as expected, was more effective the earlier it was administered after ingestion; 30 g charcoal was sufficient to prevent the absorption of 200 mg flecainide when taking simultaneously. Charcoal administration ninety minutes following flecainide ingestion also significantly decreased the amount of flecainide absorbed at four and six hours compared with control [44]. We are not aware of studies analyzing multiple dose charcoal for flecainide poisoning.

In this circumstance, we opted not to administer charcoal. The most recently administered flecainide had been dosed correctly, and at that point more than three hours had passed since ingestion. The quantity of flecainide that could be potentially prevented from absorption at that point appeared negligible. Additionally, we are not aware of any studies suggesting that charcoal can remove previously absorbed flecainide into the gastrointestinal lumen.

### What Evidence is there for the Effectiveness of Sodium Bicarbonate Administration to Treat Flecainide-Induced Cardiac Toxicity?

Multiple levels of evidence support the effectiveness of sodium bicarbonate administration to antagonize the sodium channel blocking effects of flecainide. In an in vitro model, increasing extracellular sodium reversed the effect of flecainide on the rapid inward sodium influx during phase 0 of the cardiac action potential [45]. Another in vitro model demonstrated that both alkalinization and increased extracellular sodium concentration contributed to antagonizing the effect of flecainide on sodium influx during phase 0 [46]. In a rat model of flecainide toxicity, the administration of intravenous sodium bicarbonate was effective in partially reversing flecainide-induced QRS prolongation [47]. In a canine model of flecainide toxicity, administration of sodium bicarbonate was effective in reversing QRS prolongation and treating ventricular dysrhythmias [48].

In many human cases of flecainide toxicity, administration of intravenous sodium bicarbonate appears to have been effective to narrow the QRS duration and/or reverse ventricular dysrhythmias. The preponderance of published evidence suggests that intravenous sodium bicarbonate administration should be the cornerstone of treatment [2, 7, 10, 13, 34, 35, 49–55]. Even in cases of severe toxicity, IV sodium bicarbonate has been the exclusive or predominant agent administered that appeared effective in reversing toxicity.

The beneficial effects of intravenous sodium bicarbonate generally manifest within minutes of administration and the clinical response can be dramatic. For example, an 18-day-old in cardiogenic shock from a wide complex tachycardia converted to a hemodynamically stable, narrow complex tachycardia after successive administration of two 10 mEq (5 mEq/kg) boluses [2]. In one adult case, intravenous sodium bicarbonate restored spontaneous circulation by rapidly terminating flecainide-induced ventricular fibrillation [53].

It has been noted by authors that, at least in certain published cases, IV sodium bicarbonate administration may have been inadequate, resulting in use of more invasive measures such as ECMO [2, 55].

### Can the Administration of Intravenous Sodium Bicarbonate have any Adverse Effect on the Elimination of Flecainide, and if so, how should this be Incorporated into Management?

The administration of intravenous sodium bicarbonate could potentially decrease the elimination of flecainide due to the effects of sodium bicarbonate on urine pH. Studies have demonstrated that the plasma elimination of flecainide decreases with increasing urinary pH. The pharmacokinetics of flecainide have been studied in patients administered agents to either acidify or alkalinize their urine. For alkalinization, sodium bicarbonate was administered orally, prior to, and following, a therapeutic oral dose of flecainide. This alkalinization was found to decrease elimination of flecainide compared with controls and subjects who had acidification performed [56, 57].

The clinical relevance of this effect in the setting of significant flecainide toxicity, in which aggressive administration of intravenous sodium bicarbonate is the primary therapy, remains unclear. Aggressive and appropriate treatment of flecainide-induced life-threatening cardiac toxicity would clearly take precedence over concerns of potentially prolonging the elimination of flecainide. One could consider the utilization of hypertonic saline if decreasing flecainide elimination was a concern, although there is limited evidence to support the use of hypertonic saline for the treatment of flecainide toxicity generally [58]. However, as detailed previously, the presence of bicarbonate, at least in an in vitro model of flecainide toxicity, contributed to sodium channel antagonism [46].

### Is there a Role for Intravenous Lipid Emulsion in the Treatment of Flecainide Toxicity?

Multiple case reports have detailed the administration of intravenous lipid emulsion, typically following the initial administration of sodium bicarbonate, in the setting of flecainide toxicity. In some of these cases lipid emulsion appeared to have been effective [6, 52, 59, 60].

Flecainide is considered highly lipophilic, making it a promising theoretical target for lipid emulsion therapy [61, 62]. We are aware of one animal study that evaluated the effect of intravenous lipid emulsion with flecainide toxicity. In a rabbit model, intravenous lipid emulsion was compared to hypertonic sodium bicarbonate and was found to be equivalent for the primary endpoint of mean arterial pressure at fifteen minutes and QRS duration at any timepoint. Interestingly, no increase was found in the blood concentrations of flecainide in the lipid emulsion group, suggesting that the proposed ‘lipid sink’ mechanism of lipid emulsion did not significantly contribute to the therapeutic effect [61–63]. How to clinically utilize case reports suggesting efficacy and this animal model in which lipid emulsion was compared with hypertonic sodium bicarbonate remains unclear. However, as with other highly lipophilic drugs, lipid emulsion should be considered a potential adjunctive treatment of toxicity refractory to standard accepted treatments [37].

### What other Interventions beyond Intravenous Sodium Bicarbonate and Lipid Emulsion Administration should be Considered in Cases of Severe Flecainide Toxicity?

Although the efficacy of intravenous sodium bicarbonate is well established, it is not a panacea. Life-threatening toxicity despite aggressive sodium bicarbonate administration can occur. Notably, a 33-year-old developed ventricular tachycardia leading to cardiac arrest and initiation of extracorporeal circulatory support hours after ingestion despite treatment with a total of 850 mEq bolus and a 300 mEq per hour infusion of intravenous sodium bicarbonate [64].

Other pharmacological interventions beyond sodium bicarbonate and lipid emulsion that have been temporally associated with improvement in case reports include amiodarone, lidocaine, and magnesium [6, 65–69]. Isoproterenol has successfully reduced flecainide’s toxic electrophysiologic effects in experimental studies, however the clinical evidence available is limited and inconclusive [6, 52, 70, 71].

Use of non-pharmacological interventions has been reported as well. Multiple cases have been published demonstrating successful use of mechanical circulatory support devices, namely VA-ECMO, intra-aortic balloon pump, and cardiopulmonary bypass [6, 7, 8, 20, 41, 72–83]. In particular, successful resuscitation with VA-ECMO was reported several times, with broad recommendations from authors in cases of refractory shock [7, 8, 20, 41, 74–81]. This parallels the rising prominence of VA-ECMO use in medical toxicology generally for refractory cardiotoxic poisonings [84, 85].

Cardiac pacing has a limited clinical role due to drug-induced difficulties with electrical capture [41, 66, 75, 77, 79, 86–89]. A proposed mechanism is that flecainide increases the threshold for electrical capture via toxic prolongation of the refractory period [84–86]. This effect also extends to existing permanent pacemakers [75, 87–90]. Only two reports were identified where transvenous pacing was reportedly successful [91, 92]. Similarly, electrical cardioversion and defibrillation may be less effective, although it is still advisable to follow relevant standard guidelines [41, 53, 66]. While the mechanism for this is unclear, experimental evidence in animals suggests flecainide increases the electrical threshold for successful defibrillation [93].

Extracorporeal removal of flecainide would not be expected to be beneficial based on its intrinsic properties and pharmacokinetics. Flecainide has a large volume of distribution and, as noted previously, is highly lipophilic, making it a poor hemodialysis candidate [61, 62, 94, 95]. In a study of ESRD patients, less than 1% of an oral flecainide dose was removed with routine hemodialysis [96]. Consequently, hemodialysis is not recommended [94]. Case reports of hemofiltration are consistent with minimal removal of flecainide [97, 98]. Case reports of hemoperfusion are mixed. Two cases described negligible clearance of flecainide by charcoal hemoperfusion and three cases described improved drug clearance with hemodialysis plus hemoperfusion, two using a charcoal cartridge and one using the CytoSorb® (Cytosorbents Europe GmbH, Berlin, Germany) cartridge, although with unclear clinical benefit [98–101].

## Case Continuation

*The medical toxicology service was consulted (authors JS, AS) and recommended admission to the neonatal intensive care unit for close monitoring on telemetry. We did not recommend administration of activated charcoal or sodium bicarbonate, nor any other interventions at the time of admission. The patient had most recently ingested a therapeutic flecainide dose 2.5 h prior to presentation. As flecainide is rapidly absorbed, with peak plasma concentrations in 1 to 3 h, we felt the patient’s presentation likely represented post peak toxicity and we did not anticipate clinical worsening* [102]. *Further, it would be challenging to administer activated charcoal to a neonate and require placement of a nasogastric tube, which would take significant additional time and thus be of limited clinical utility.*

*We did recommend having intravenous sodium bicarbonate immediately available in the unlikely event further QRS prolongation were to occur. Following admission, the primary team opted to trial a 1 milliequivalent per kilogram bolus of sodium bicarbonate 4.2%, resulting in no noted effect on the QRS duration, so no additional doses were given.*

*The serum flecainide concentration drawn while in the emergency department, approximately 6.5 h following the last dose of flecainide, was 1.6 µg/ml (therapeutic range 0.20–1.00 µg/ml).*

### Is the Flecainide Concentration Consistent with ECG Findings and the Dosing Error Described?

As flecainide impacts cardiac conduction in an established, dose-dependent manner, the ECG findings described above appeared consistent with the supratherapeutic concentration of flecainide measured [4, 33, 38, 74, 103–106]. The flecainide concentration also appeared to be consistent with the supratherapeutic dosing. As described above, there did not appear to be any potentially confounding factors such as CYP2D6 expression, drug interaction, electrolyte abnormality, hepatic or renal dysfunction. For this reason, analysis of the patients CYP2D6 expression was not performed. Additionally, analysis of the dispensed flecainide bottle was not performed as the toxicity appeared consistent with the labelling.

## Case Conclusion

**Fig. 3:**
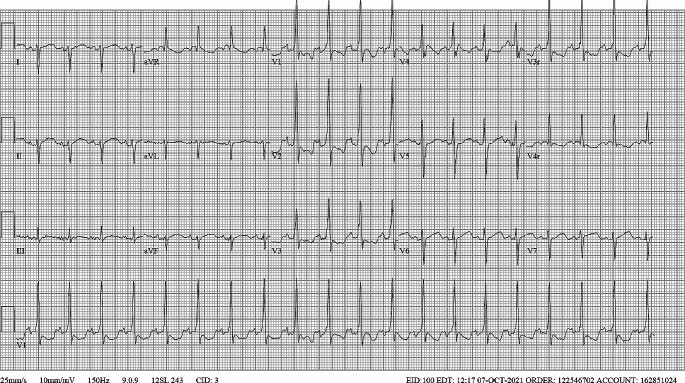
ECG at the time of hospital discharge

*The electrocardiographic abnormalities normalized by sixteen hours after admission (*Fig. 3*). The patient was discharged less than 24 h after initial presentation with instructions to restart flecainide at the intended concentration that afternoon and continue on propranolol. As of 6 months follow up, the patient has had no recurrent episodes of toxicity or SVT. Propranolol was discontinued 2 months after presentation and flecainide 2 months after that. The patient’s family then moved and the patient was subsequently lost to follow up.*

### What has Been Suggested to help Prevent Flecainide Dosing Errors in the Pediatric Population?

Suggestions have included having a standardized concentration available in both inpatient and outpatient settings. It has been recommended that prescribers order flecainide in terms of the total dose but that the label detail quantity in terms of total volume [5]. Unambiguous, perhaps color-coded labeling, particularly when a patient is on multiple drugs, has also been suggested [49].

## References

[1] Boehringer Ingelheim, Tablets FLECAINIDEACETATE, Accessed USP. May 30, 2023. Revised September, 2014. https://docs.boehringer-ingelheim.com/Prescribing%20Information/PIs/Roxane/Flecainide%20Acetate/Flecainide%20Acetate%20Tablets.pdf.

[2] Jang DH, Hoffman RS, Nelson LS. A case of near-fatal flecainide overdose in a neonate successfully treated with sodium bicarbonate. J Emerg Med. 2013;44(4):781–3. 22981658 10.1016/j.jemermed.2012.07.050 PMC3985060

[3] Andrikopoulos GK, Pastromas S, Tzeis S, Flecainide. Current status and perspectives in arrhythmia management. World J Cardiol. 2015;7(2):76–85. 10.4330/wjc.v7.i2.76 PMC4325304 25717355

[4] Tamargo J, Le Heuzey JY, Mabo P. Narrow therapeutic index drugs: a clinical pharmacological consideration to flecainide. Eur J Clin Pharmacol. 2015;71(5):549–67. 25870032 10.1007/s00228-015-1832-0 PMC4412688

[5] Shastay A. Life-threatening errors with Flecainide suspension in children. Home Healthc Now. 2017;35(1):50–1. 27923000 10.1097/NHH.0000000000000474

[6] Karmegaraj B, Menon D, Prabhu MA, Vaidyanathan B. Flecainide toxicity in a preterm neonate with permanent junctional reciprocating tachycardia. Ann Pediatr Cardiol. 2017 Sep-Dec;10(3):288–92. 10.4103/apc.APC_31_17 PMC5594942 28928617

[7] Kwan D, Vohra R, Dyer JE, Dornhoffer P. An infant with a heartbreaking medication error. Pediatr Emerg Care. 2014;30(12):e1–5. 25469608 10.1097/PEC.0000000000000320

[8] Bhimani SA, Rao S, Nadorlik H, Saarel EV, Aziz PF. Flecainide toxicity in renal tubular acidosis type IV treated with extracorporeal membrane oxygenation. HeartRhythm Case Rep. 2020;6(5):287–9. 32461897 10.1016/j.hrcr.2020.02.001 PMC7244634

[9] Stuart AG, Wren C, Bain HH. Is there a genetic factor in flecainide toxicity? BMJ. 1989;298(6666):117–8. 2493285 10.1136/bmj.298.6666.117-b PMC1835442

[10] Close BR, Banks CJ. Pediatric flecainide toxicity from a double dose. Am J Emerg Med. 2012;30(9):e20951–2. 10.1016/j.ajem.2012.01.005 22386360

[11] Bajaj S, Tullu MS, Khan Z, Agrawal M. When potion becomes poison! A case report of flecainide toxicity. J Postgrad Med. 2017;63(4):265–7. 28272074 10.4103/0022-3859.201422 PMC5664873

[12] Rasheed A, Simpson J, Rosenthal E. Neonatal ECG changes caused by supratherapeutic flecainide following treatment for fetal supraventricular tachycardia. Heart. 2003;89(4):470. 12639886 10.1136/heart.89.4.470-a PMC1769267

[13] Barot. A case of neonatal single twin flecainide toxicity after therapeutic in utero exposure for fetal SVT.

[14] Falk RH. Flecainide-induced ventricular tachycardia and fibrillation in patients treated for atrial fibrillation. Ann Intern Med. 1989;111(2):107–11. 2500880 10.7326/0003-4819-111-2-107

[15] Lavalle C, Trivigno S, Vetta G, Magnocavallo M, Mariani MV, Santini L, Forleo GB, Grimaldi M, Badagliacca R, Lanata L, Ricci RP. Flecainide in Ventricular arrhythmias: from old myths to New perspectives. J Clin Med. 2021;10(16):3696. 34441994 10.3390/jcm10163696 PMC8397118

[16] Teh LK, Bertilsson L. Pharmacogenomics of CYP2D6: molecular genetics, interethnic differences and clinical importance. Drug Metab Pharmacokinet. 2012;27(1):55–67. 22185816 10.2133/dmpk.DMPK-11-RV-121

[17] Thomas CD, Johnson JA. Pharmacogenetic factors affecting β-blocker metabolism and response. Expert Opin Drug Metab Toxicol. 2020;16(10):953–64. 32726152 10.1080/17425255.2020.1803279 PMC7606773

[18] Gross AS, Mikus G, Fischer C, Eichelbaum M. Polymorphic flecainide disposition under conditions of uncontrolled urine flow and pH. Eur J Clin Pharmacol. 1991;40(2):155–62. 1906003 10.1007/BF00280070

[19] Doki K, Homma M, Kuga K, Kusano K, Watanabe S, Yamaguchi I, Kohda Y. Effect of CYP2D6 genotype on flecainide pharmacokinetics in Japanese patients with supraventricular tachyarrhythmia. Eur J Clin Pharmacol. 2006;62(11):919–26. 16944116 10.1007/s00228-006-0188-x

[20] Poh BH, Lee JH, Abdul Haium AA, Choo TLJ. Complete Heart Block secondary to Flecainide Toxicity: is it time for CYP2D6 genotype testing? Pediatrics. 2020;146(1):e20192608. 32561613 10.1542/peds.2019-2608

[21] O’Hara K, Wright IM, Schneider JJ, Jones AL, Martin JH. Pharmacokinetics in neonatal prescribing: evidence base, paradigms and the future. Br J Clin Pharmacol. 2015;80(6):1281–8. 26256466 10.1111/bcp.12741 PMC4693494

[22] Beckmann J, Hertrampf R, Gundert-Remy U, Mikus G, Gross AS, Eichelbaum M. Is there a genetic factor in flecainide toxicity? BMJ. 1988;297(6659):1316. 3144374 10.1136/bmj.297.6659.1316 PMC1834956

[23] Zhou SF. Polymorphism of human cytochrome P450 2D6 and its clinical significance: part I. Clin Pharmacokinet. 2009;48(11):689–723. 19817501 10.2165/11318030-000000000-00000

[24] Haefeli WE, Bargetzi MJ, Follath F, Meyer UA. Potent inhibition of cytochrome P450IID6 (debrisoquin 4-hydroxylase) by flecainide in vitro and in vivo. J Cardiovasc Pharmacol. 1990;15(5):776–9. 1692938 10.1097/00005344-199005000-00013

[25] Shea P, Lal R, Kim SS, Schechtman K, Ruffy R. Flecainide and amiodarone interaction. J Am Coll Cardiol. 1986;7(5):1127–30. 3958371 10.1016/S0735-1097(86)80234-0

[26] Leclercq JF, Denjoy I, Mentré F, Coumel P. Flecainide acetate dose-concentration relationship in cardiac arrhythmias: influence of heart failure and amiodarone. Cardiovasc Drugs Ther. 1990;4(4):1161–5. 2128031 10.1007/BF01856514

[27] Tsao YY, Gugger JJ. Delirium in a patient with toxic flecainide plasma concentrations: the role of a pharmacokinetic drug interaction with paroxetine. Ann Pharmacother. 2009;43(7):1366–9. 19531695 10.1345/aph.1M067

[28] US Food and Drug Administration. Drug Development and Drug Interactions| Table of Substrates, Inhibitors and Inducers. Access May 30. 2023. Revised August 24, 2022. https://www.fda.gov/drugs/drug-interactions-labeling/drug-development-and-drug-interactions-table-substrates-inhibitors-and-inducers#table3-2.

[29] McQuinn RL, Pentikäinen PJ, Chang SF, Conard GJ. Pharmacokinetics of flecainide in patients with cirrhosis of the liver. Clin Pharmacol Ther. 1988;44(5):566–72. 3141098 10.1038/clpt.1988.195

[30] Braun J, Kollert JR, Becker JU. Pharmacokinetics of flecainide in patients with mild and moderate renal failure compared with patients with normal renal function. Eur J Clin Pharmacol. 1987;31(6):711–4. 3104059 10.1007/BF00541300

[31] Ting SM, Lee D, Maclean D, Sheerin NS. Paranoid psychosis and myoclonus: flecainide toxicity in renal failure. Cardiology. 2008;111(2):83–6. 18376118 10.1159/000119694

[32] Ramhamadany E, Mackenzie S, Ramsdale DR. Dysarthria and visual hallucinations due to flecainide toxicity. Postgrad Med J. 1986;62(723):61–2. 3099275 10.1136/pgmj.62.723.61 PMC2418559

[33] Smith A, Gerasimon G. An electrocardiographic series of flecainide toxicity. Indian Pacing Electrophysiol J 2019 Mar-Apr;19(2):75–8. 10.1016/j.ipej.2018.11.012 PMC6450827 30502382

[34] Subedi R, Dean RK, Chaudhary A, Szombathy T. Flecainide toxicity in renal failure. Proc (Bayl Univ Med Cent). 2018;31(3):328–30. 29904301 10.1080/08998280.2018.1463042 PMC5997054

[35] Newson JM, Santos CD, Walters BL, Todd BR. The case of Flecainide Toxicity: what to look for and how to treat. J Emerg Med. 2020;59(2):e43–7. 32536493 10.1016/j.jemermed.2020.04.052

[36] Mikus G, Gross AS, Beckmann J, Hertrampf R, Gundert-Remy U, Eichelbaum M. The influence of the sparteine/debrisoquin phenotype on the disposition of flecainide. Clin Pharmacol Ther. 1989;45(5):562–7. 2498026 10.1038/clpt.1989.73

[37] Ghataoura R, Patil S. Flecainide toxicity: a presentation to the emergency department with literature review. BMJ Case Rep. 2020;13(2):e232691. 32114494 10.1136/bcr-2019-232691 PMC7050315

[38] Morganroth J, Horowitz LN. Flecainide: its proarrhythmic effect and expected changes on the surface electrocardiogram. Am J Cardiol. 1984;53(5):B89–94. 10.1016/0002-9149(84)90509-5 6695821

[39] Estes NA 3rd, Garan H, Ruskin JN. Electrophysiologic properties of flecainide acetate. Am J Cardiol. 1984;53(5):B26–9. 10.1016/0002-9149(84)90498-3 6695816

[40] Winkelmann BR, Leinberger H. Life-threatening flecainide toxicity. A pharmacodynamic approach. Ann Intern Med. 1987;106(6):807–14. 3107447 10.7326/0003-4819-106-6-807

[41] Vu NM, Hill TE, Summers MR, Vranian MN, Faulx MD. Management of life-threatening flecainide overdose: a case report and review of the literature. HeartRhythm Case Rep. 2015;2(3):228–31. 28491675 10.1016/j.hrcr.2015.12.013 PMC5419747

[42] Gentzkow GD, Sullivan JY. Extracardiac adverse effects of flecainide. Am J Cardiol. 1984;53(5):B101–5. 10.1016/0002-9149(84)90511-3 6695813

[43] Köppel C, Oberdisse U, Heinemeyer G. Clinical course and outcome in class IC antiarrhythmic overdose. J Toxicol Clin Toxicol. 1990;28(4):433–44. 2176700 10.3109/15563659009038586

[44] Nitsch J, Köhler U, Neyses L, Lüderitz B. Inhibition of flecainide absorption by activated charcoal. Am J Cardiol. 1987;60(8):753. 3116835 10.1016/0002-9149(87)90405-X

[45] Ranger S, Sheldon R, Fermini B, Nattel S. Modulation of flecainide’s cardiac sodium channel blocking actions by extracellular sodium: a possible cellular mechanism for the action of sodium salts in flecainide cardiotoxicity. J Pharmacol Exp Ther. 1993;264(3):1160–7. 8383739

[46] Bou-Abboud E, Nattel S. Relative role of alkalosis and sodium ions in reversal of class I antiarrhythmic drug-induced sodium channel blockade by sodium bicarbonate. Circulation. 1996;94(8):1954–61. 8873674 10.1161/01.CIR.94.8.1954

[47] Keyler DE, Pentel PR. Hypertonic sodium bicarbonate partially reverses QRS prolongation due to flecainide in rats. Life Sci. 1989;45(17):1575–80. 2555639 10.1016/0024-3205(89)90424-4

[48] Salerno DM, Murakami MM, Johnston RB, Keyler DE, Pentel PR. Reversal of flecainide-induced ventricular arrhythmia by hypertonic sodium bicarbonate in dogs. Am J Emerg Med. 1995;13(3):285–93. 7755819 10.1016/0735-6757(95)90201-5

[49] D’Alessandro LC, Rieder MJ, Gloor J, Freeman D, Buffo-Sequiera I. Life-threatening flecainide intoxication in a young child secondary to medication error. Ann Pharmacother. 2009;43(9):1522–7. 19671803 10.1345/aph.1L549

[50] Lovecchio F, Berlin R, Brubacher JR, Sholar JB. Hypertonic sodium bicarbonate in an acute flecainide overdose. Am J Emerg Med. 1998;16(5):534–7. 9725977 10.1016/S0735-6757(98)90013-4

[51] Goldman MJ, Mowry JB, Kirk MA. Sodium bicarbonate to correct widened QRS in a case of flecainide overdose. J Emerg Med. 1997 Mar-Apr;15(2):183–6. 10.1016/s0736-4679(96)00345-9 9144059

[52] Gardner Yelton SE, Leonard JB, de la Uz CM, Wadia RS, Barnes SS. Flecainide Toxicity secondary to Accidental Overdose: A Pediatric Case Report of two brothers. Case Rep Crit Care. 2021;2021:6633859. 34094603 10.1155/2021/6633859 PMC8140826

[53] Jung HW, Kwak JJ, Namgung J. Flecainide-Induced Torsade De Pointes successfully treated with intensive pharmacological therapy. Int J Arrhythm. 2016;17(2):97–102. 10.18501/arrhythmia.2016.018

[54] Rognoni A, Bertolazzi M, Peron M, Macciò S, Cameroni GT, Gratarola A, Rognoni G. Electrocardiographic changes in a rare case of flecainide poisoning: a case report. Cases J. 2009;2:9137. 20062654 10.1186/1757-1626-2-9137 PMC2803934

[55] Devin R, Garrett P, Anstey C. Managing cardiovascular collapse in severe flecainide overdose without recourse to extracorporeal therapy. Emerg Med Australas. 2007;19(2):155–9. 17448102 10.1111/j.1742-6723.2006.00909.x

[56] Muhiddin KA, Johnston A, Turner P. The influence of urinary pH on flecainide excretion and its serum pharmacokinetics. Br J Clin Pharmacol. 1984;17(4):447–51. 6326790 10.1111/j.1365-2125.1984.tb02370.x PMC1463395

[57] Johnston A, Warrington S, Turner P. Flecainide pharmacokinetics in healthy volunteers: the influence of urinary pH. Br J Clin Pharmacol. 1985;20(4):333–8. 4074602 10.1111/j.1365-2125.1985.tb05073.x PMC1400890

[58] Severe Flecainide Toxicity with Cardiac Arrest. Treated with 3% hypertonic saline in addition to Standard Sodium Bicarbonate Therapy. Laura Tortora, Joshua Canning ACMT 2019 Annual Scientific Meeting abstracts—San Francisco, CA. J Med Toxicol. 2019;15:53–107. 30825071 10.1007/s13181-019-00699-x PMC6441065

[59] Khatiwada P, Clark L, Khunger A, Rijal BB, Ritter J. A Case Report of Flecainide Toxicity with Review of Literature. Cureus. 2022;14(2):e22261. 35350525 10.7759/cureus.22261 PMC8933271

[60] Moussot PE, Marhar F, Minville V, Vallé B, Dehours E, Bounes V, Ducassé JL. Use of intravenous lipid 20% emulsion for the treatment of a voluntary intoxication of flecainide with refractory shock. Clin Toxicol (Phila). 2011;49(6):514. 21824065 10.3109/15563650.2011.590940

[61] Cave G, Harvey M, Quinn P, Heys D. Hypertonic sodium bicarbonate versus intravenous lipid emulsion in a rabbit model of intravenous flecainide toxicity: no difference, no sink. Clin Toxicol (Phila). 2013;51(5):394–7. 23700986 10.3109/15563650.2013.794282

[62] Ellsworth H, Stellpflug SJ, Cole JB, Dolan JA, Harris CR. A life-threatening flecainide overdose treated with intravenous fat emulsion. Pacing Clin Electrophysiol. 2013;36(3):e87–9. 22882363 10.1111/j.1540-8159.2012.03485.x

[63] Fettiplace MR, Weinberg G. The mechanisms underlying lipid resuscitation therapy. Reg Anesth Pain Med. 2018;43(2):138–49. 29356774 10.1097/AAP.0000000000000719

[64] Brumfield E, Bernard KR, Kabrhel C. Life-threatening flecainide overdose treated with intralipid and extracorporeal membrane oxygenation. Am J Emerg Med. 2015;33(12):e18403–5. 10.1016/j.ajem.2015.04.012 25921969

[65] Siegers A, Board PN. Amiodarone used in successful resuscitation after near-fatal flecainide overdose. Resuscitation. 2002;53(1):105–8. 11947987 10.1016/S0300-9572(01)00503-2

[66] Wynn J, Fingerhood M, Keefe D, Maza S, Miura D, Somberg JC. Refractory ventricular tachycardia with flecainide. Am Heart J. 1986;112(1):174–5. 3728275 10.1016/0002-8703(86)90699-X

[67] Bauman JL, Gallastegui J, Tanenbaum SR, Hariman RJ. Flecainide-induced sustained ventricular tachycardia successfully treated with lidocaine. Chest. 1987;92(3):573–5. 3113836 10.1378/chest.92.3.573

[68] Williamson DG, Sinha A, Frost I, Singh VK. Management of persistent wide QRS in flecainide overdose with magnesium sulphate. Emerg Med J. 2010;27(6):487–8. 20562156 10.1136/emj.2009.081075

[69] Hanley NA, Bourke JP, Gascoigne AD. Survival in a case of life-threatening flecainide overdose. Intensive Care Med. 1998;24(7):740–2. 9722048 10.1007/s001340050655

[70] Avitall B, Hare JW, Tchou P, Jazayeri M, Akhtar M. Flecainide toxicity: reversal of drug effects by isoproterenol infusion. J Cardiovasc Electrophys. 1991;2(5):431–40. 10.1111/j.1540-8167.1991.tb01343.x

[71] Manolis AS, Estes NA 3rd. Reversal of electrophysiologic effects of flecainide on the accessory pathway by isoproterenol in the Wolff-Parkinson-White syndrome. Am J Cardiol. 1989;64(3):194–8. 2500840 10.1016/0002-9149(89)90456-6

[72] Timperley J, Mitchell AR, Brown PD, West NE. Flecainide overdose–support using an intra-aortic balloon pump. BMC Emerg Med. 2005;5:10. 16343338 10.1186/1471-227X-5-10 PMC1325039

[73] Van Reet B, Dens J. Auto-intoxication with flecainide and quinapril: ECG-changes, symptoms and treatment. Acta Cardiol. 2006;61(6):669–72. 17205927 10.2143/AC.61.6.2017969

[74] Valentino MA, Panakos A, Ragupathi L, Williams J, Pavri BB. Flecainide Toxicity: a case report and systematic review of its electrocardiographic patterns and management. Cardiovasc Toxicol. 2017;17(3):260–6. 27435408 10.1007/s12012-016-9380-0

[75] Nadel J, Kumarasinghe G, Subbiah R. The Unpaceable Heart. JACC Case Rep. 2020;2(4):595–7. 34317301 10.1016/j.jaccas.2020.02.010 PMC8298662

[76] Hantson P, Wuidart C, Haufroid V. Severe and prolonged flecainide intoxication treated by extracorporeal life support: possible role of cytochrome P450 2D6 polymorphism? Clin Toxicol (Phila). 2019;57(7):672–3. 30757931 10.1080/15563650.2018.1542703

[77] Reynolds JC, Judge BS. Successful treatment of flecainide-induced cardiac arrest with extracorporeal membrane oxygenation in the ED. Am J Emerg Med. 2015;33(10):e15421–2. 10.1016/j.ajem.2015.07.054 26299692

[78] Mandawat A, McCullough SA, Gilstrap LG, Yeh RW. Successful treatment of flecainide overdose with sustained mechanical circulatory support. HeartRhythm Case Rep. 2015;1(3):137–40. 28491532 10.1016/j.hrcr.2015.01.003 PMC5418611

[79] Auzinger GM, Scheinkestel CD. Successful extracorporeal life support in a case of severe flecainide intoxication. Crit Care Med. 2001;29(4):887–90. 11373489 10.1097/00003246-200104000-00041

[80] Sivalingam SK, Gadiraju VT, Hariharan MV, Atreya AR, Flack JE, Aziz H. Flecainide toxicity–treatment with intravenous fat emulsion and extra corporeal life support. Acute Card Care. 2013;15(4):90–2. 24200150 10.3109/17482941.2013.841949

[81] Vivien B, Deye N, Mégarbane B, Marx JS, Leprince P, Bonnet N, Roussin F, Jacob L, Pavie A, Baud FJ, Carli P. Extracorporeal life support in a case of fatal flecainide and betaxolol poisoning allowing successful cardiac allograft. Ann Emerg Med. 2010;56(4):409–12. 20172626 10.1016/j.annemergmed.2010.01.021

[82] Corkeron MA, van Heerden PV, Newman SM, Dusci L. Extracorporeal circulatory support in near-fatal flecainide overdose. Anaesth Intensive Care. 1999;27(4):405–8. 10470398 10.1177/0310057X9902700413

[83] Yasui RK, Culclasure TF, Kaufman D, Freed CR. Flecainide overdose: is cardiopulmonary support the treatment? Ann Emerg Med. 1997;29(5):680–2. 9140253 10.1016/S0196-0644(97)70257-9

[84] Ng M, Wong ZY, Ponampalam R. Extracorporeal cardio-pulmonary resuscitation in poisoning: a scoping review article. Resusc Plus. 2023;13:100367. 36860990 10.1016/j.resplu.2023.100367 PMC9969255

[85] Upchurch C, Blumenberg A, Brodie D, MacLaren G, Zakhary B, Hendrickson RG. Extracorporeal membrane oxygenation use in poisoning: a narrative review with clinical recommendations. Clin Toxicol (Phila). 2021;59(10):877–87. 34396873 10.1080/15563650.2021.1945082

[86] Hellestrand KJ, Burnett PJ, Milne JR, Bexton RS, Nathan AW, Camm AJ. Effect of the antiarrhythmic agent flecainide acetate on acute and chronic pacing thresholds. Pacing Clin Electrophysiol. 1983;6(5 Pt 1):892–9. 6195608 10.1111/j.1540-8159.1983.tb04410.x

[87] Manley-Casco D, Crass S, Alqusairi R, Girard S. Flecainide toxicity with high pacemaker capture thresholds and associated takotsubo syndrome. BMJ Case Rep. 2021;14(8):e243326. 34362750 10.1136/bcr-2021-243326 PMC8351479

[88] Apps A, Miller CP, Fellows S, Jones M. Cardiac devices with class 1 C antiarrhythmics: a potentially toxic combination. BMJ Case Rep. 2015;2015:bcr2015210598. 26286909 10.1136/bcr-2015-210598 PMC4550908

[89] Rivner H, Lambrakos LK. Flecainide Toxicity leading to loss of Pacemaker capture and Cardiac arrest. JACC Case Rep. 2021;3(4):586–90. 34317582 10.1016/j.jaccas.2020.11.030 PMC8302776

[90] Heldens M, van der Nat GAM, Melman PG. Renal failure, shock, and loss of pacemaker capture: a case of flecainide intoxication. Neth J Med. 2019;77(5):189–92. 31264585

[91] Lloyd T, Zimmerman J, Griffin GD. Irreversible third-degree heart block and pacemaker implant in a case of flecainide toxicity. Am J Emerg Med. 2013;31(9):e14181–2. 10.1016/j.ajem.2013.04.025 23810074

[92] Götz D, Pohle S, Barckow D. Primary and secondary detoxification in severe flecainide intoxication. Intensive Care Med. 1991;17(3):181–4. 1906489 10.1007/BF01704725

[93] Murakawa Y, Inoue H, Kuo TT, Sezaki K, Nakajima T, Usui M, Yamashita T, Ajiki K, Oikawa N, Sugimoto T, et al. Prolongation of intraventricular conduction time associated with fatal [correction of fetal] impairment of defibrillation efficiency during treatment with class I antiarrhythmic agents. J Cardiovasc Pharmacol. 1995;25(2):194–9. 7752644 10.1097/00005344-199502000-00003

[94] Tjandra-Maga TB, Verbesselt R, Van Hecken A, Mullie A, De Schepper PJ. Flecainide: single and multiple oral dose kinetics, absolute bioavailability and effect of food and antacid in man. Br J Clin Pharmacol. 1986;22(3):309–16. 3094570 10.1111/j.1365-2125.1986.tb02892.x PMC1401142

[95] King JD, Kern MH, Jaar BG. Extracorporeal removal of poisons and toxins. Clin J Am Soc Nephrol. 2019;14(9):1408–15. 31439539 10.2215/CJN.02560319 PMC6730523

[96] Forland SC, Burgess E, Blair AD, Cutler RE, Kvam DC, Weeks CE, Fox JM, Conard GJ. Oral flecainide pharmacokinetics in patients with impaired renal function. J Clin Pharmacol. 1988;28(3):259–67. 3129455 10.1002/j.1552-4604.1988.tb03142.x

[97] Borgeat A, Biollaz J, Freymond B, Bayer-Berger M, Chiolero R. Hemofiltration clearance of flecainide in a patient with acute renal failure. Intensive Care Med. 1988;14(3):236–7. 3132493 10.1007/BF00717997

[98] Steinmetz M, Nickenig G, Sauerbruch T, Eyer F, Rabe C. Effect of hemoperfusion on flecainide serum concentration - a case report. Clin Toxicol (Phila). 2017;55(2):153–4. 27728980 10.1080/15563650.2016.1241400

[99] Dirks E, Gieshoff B, Stahlmann R, Wehr M, Hager W. Erfolgreiche Therapie Einer Flecainid-(Tambocor-)Intoxikation–Effekt einer Hämodialyse/Hämoperfusion? [Successful treatment of flecainide (Tambocor) poisoning–effect of hemodialysis/hemoperfusion?]. Z Kardiol. 1990;79(1):54–9. 2107645

[100] De Schryver N, Hantson P, Haufroid V, Dechamps M. Cardiogenic shock in a Hemodialyzed patient on Flecainide: treatment with Intravenous Fat Emulsion, extracorporeal Cardiac Life Support, and CytoSorb® Hemoadsorption. Case Rep Cardiol. 2019;2019:1905871. 31428479 10.1155/2019/1905871 PMC6681578

[101] Wurzberger R, Witter E, Avenhaus H, Hennemann H, Becker JU. Hämoperfusion Bei Flecainidintoxikation [Hemoperfusion in flecainide poisoning]. Klin Wochenschr. 1986;64(9):442–4. 3713113 10.1007/BF01727530

[102] Lavalle C, Magnocavallo M, Straito M, Santini L, Forleo GB, Grimaldi M, Badagliacca R, Lanata L, Ricci RP. Flecainide how and when: a practical guide in supraventricular arrhythmias. J Clin Med. 2021;10(7):1456. 33918105 10.3390/jcm10071456 PMC8036302

[103] Hellestrand KJ, Bexton RS, Nathan AW, Spurrell RA, Camm AJ. Acute electrophysiological effects of flecainide acetate on cardiac conduction and refractoriness in man. Br Heart J. 1982;48(2):140–8. 7093083 10.1136/hrt.48.2.140 PMC481218

[104] Levis JT. ECG diagnosis: flecainide toxicity. Perm J 2012 Fall;16(4):53. 10.7812/tpp/12-086 PMC3523937 23251119

[105] Apfelbaum JD, Gerczynski J, Robertson WE, Richey S, Electrocardiography. Flecainide Toxic Emerg Med. 2018;50:124–6.

[106] Rognoni A, Bertolazzi M, Peron M, Macciò S, Cameroni GT, Gratarola A, Rognoni G. Electrocardiographic changes in a rare case of flecainide poisoning: a case report. Cases J. 2009;2:1–4. 20062654 10.1186/1757-1626-2-9137 PMC2803934

